# Case Report: A woman with pelvic hydatid disease

**DOI:** 10.4102/sajid.v37i1.455

**Published:** 2022-11-10

**Authors:** Nabeela Adam

**Affiliations:** 1 Department of Orthopaedic Surgery, Frere Hospital, East London, South Africa; 2 Department of Orthopaedic Surgery, Walter Sisulu University, Mthatha, South Africa

**Keywords:** hydatid disease, hydatid, pelvic hydatid, bone hydatid

## Abstract

Hydatid disease is found in many low-income countries, where humans are deemed accidental hosts. Bone hydatid disease is a rare entity as the lungs and liver are usually the target organs. However, there are currently a handful of case reports around the world with bone hydatid disease.

**Contribution:**

We present South Africa’s first case report of pelvic hydatid disease and looking to uncover some treatment options for this patient.

## Introduction

### Case presentation

A 49-year-old woman of African descent was referred to the Orthopaedic Department at Frere Hospital in East London; she had had a draining sinus over her left hip for the last five months. She was complaining of pain and difficulty walking. She was noted to be obese, with a body mass index (BMI) of 31, and she was HIV nonreactive. Her past medical history included a laparotomy in 2019 when she was found to have extensive hydatid disease in her abdomen and was then told to take an antibiotic for a year. She had no known allergies and was unemployed. On examination, it was noted that she mobilised using a walking stick and could not walk long distances due to severe pain. She used a wheelchair. On examination of her abdomen, a large 10 cm scar was noted over the left inguinal area, and over her left buttock area there was a 0.5 cm wound that was draining a yellowish-brown fluid. The general surgeons took the patient to the theatre for another look at the abdomen and found that there were several cysts that were originating from the left iliac wing bone as well as the abdomen. The patient had refused any further surgical intervention and was not keen on trying new and alternative treatment options. She has also not been followed up, as she has missed her follow-up appointments.

**FIGURE 1:**
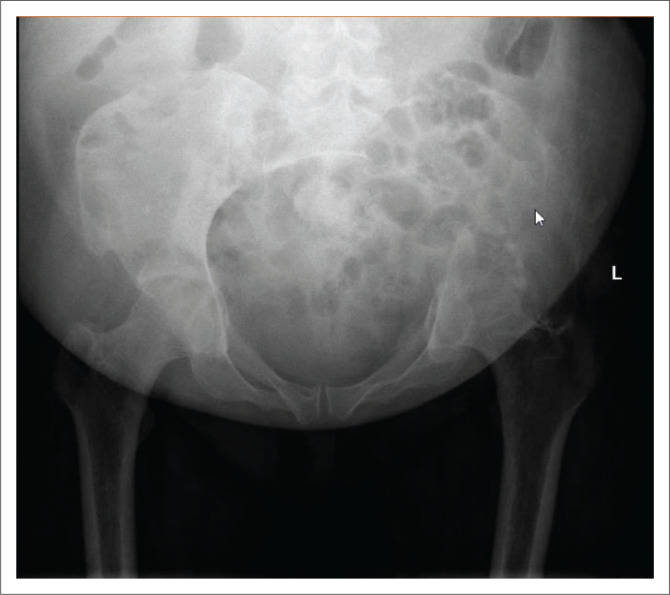
An X-ray demonstrated an absent left hemipelvis.

**FIGURE 2:**
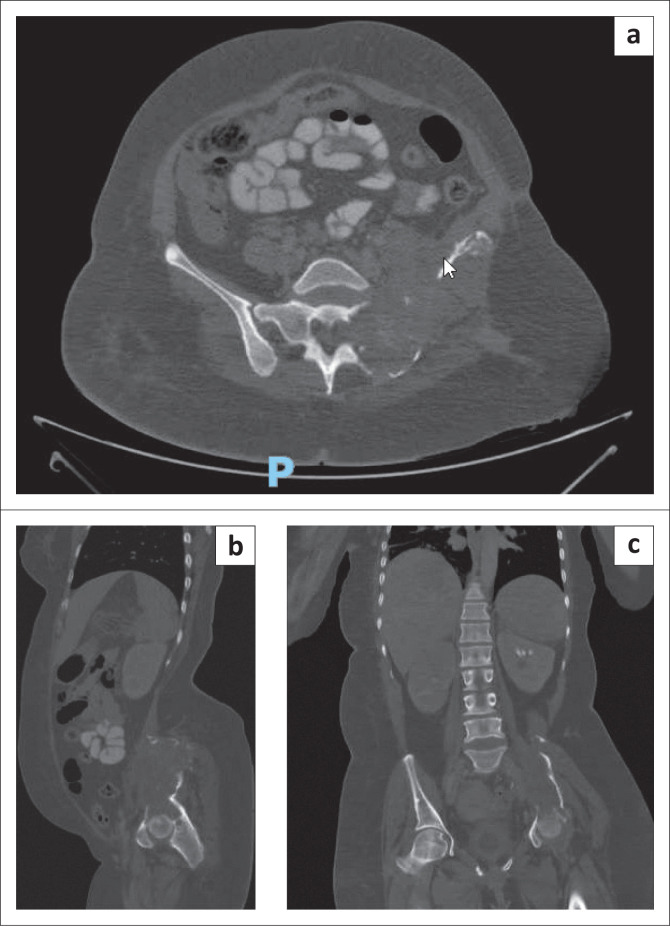
(a, b, c) Computerised tomography images.

Figure 1 is an X-ray of the pelvis and Figure 2a, b and c is computerised tomography (CT) images demonstrating the entire left hemipelvis which was replaced by a large cystic mass.

Hydatid disease is a zoonosis that is caused by parasites that belong to the *Echinococcus* species of the Taeniidae family of cestodes. *Echinococcus* cysts are caused by the larval stage of dog tapeworms, and the disease is found in many low-income areas on all continents except Antarctica.^1^ The life cycle involves two hosts: canines are the definitive hosts, while various herbivores are the usual intermediate hosts. Humans are accidental intermediate hosts. Hydatid cysts are commonly found in the liver and lung, and in rare cases they are found in the bone, spleen and breast.^2^ Humans can be infected by any of the three kinds of echinococcosis: cystic (produced by *E. granulosus*), alveolar (caused by *E. multilocularis*) and polycystic (induced by either *E. vogeli* or *E. oligarthrus*). *E. granulosus* and *E. multilocularis* most frequently affect human hosts.^3^

The definitive hosts are dogs, foxes and other carnivores. Intermediate hosts for *E. granulosus* are sheep, cattle and other herbivores that harbour the hydatid cysts. Dogs eat the cysts containing the larval tapeworms, called protoscoleces. These scoleces attach to the intestine of the dog, and then the adult tapeworms will form, shedding eggs in the faeces when mature. The eggs are then eaten by the intermediate host, such as the sheep. Once swallowed, an egg will release an oncosphere, which penetrates the intestine and is carried in the circulation to form the hydatid cyst in the liver, lung or elsewhere. Humans are accidental hosts, infected by ingesting food, water or soil that is contaminated with these eggs. Hydatid cysts most commonly affect the liver or lungs of the humans.^3^ Cystic echinococcosis is responsible for 95% of all hydatid cyst cases in humans.^4^

Medical treatment options for hydatid disease are the anthelminthics albendazole and praziquantel.^5^ In more advanced cases, surgical debridement is necessary when extensive disease is found or disease not responding to medical treatment. Several case studies have been reported across the world, with many citing difficulties in treating patients with pelvic hydatid disease.

Another treatment that is increasingly becoming popular is PAIR (puncture, aspiration, injection and re-aspiration); this is a novel treatment that was initially described to treat liver hydatid disease in 374 patients.^6^ It was a great success story because it was a low-cost treatment option with minimal surgical risks.

The problem arises when patients have resistant disease or recurrence of disease. What are the treatment options in such cases? What are the treatment options when bone disease is present?

A case study in which sternal hydatid disease was treated with radiation therapy concluded that while it did work in their case study, it did provide an alternative treatment option in what is already a difficult area to treat.^5^ In 2017, a case report of pelvic hydatid disease was successfully treated together with surgical debridement and anthelminthic therapy.^3^ Another case report from Karachi used a limb salvage procedure in which a hemipelvectomy together with resection of the proximal femur was done; together with postoperative anthelminthics, the patient at the 5-year follow-up was reported to finally be without disease and walking without assistance.^7^ In 2009, another case report of a patient in Italy with pelvic disease was offered a total hip replacement but had two revision surgeries and is now reported to be pain-free, and the prosthesis is correctly positioned and stable.^8^

South Africa has a number of limited case studies on hydatid disease but none specifically on bone hydatid disease. A hydatid disease study with significant impact is one from 2019, which showed that 50% of its 22 patients were found to have HIV co-infection.^9^ This is an interesting finding and is something that should be investigated in the future, because Southern Africa has the highest incidence of HIV in the world.^10^

The incidence of bone hydatid disease is low, and it is nonetheless challenging to treat, particularly if it affects places that are challenging to reach during surgery. Because the prevalence of bone hydatid disease in South Africa is not known, more information is required to study this condition there. Given that this patient’s condition has significantly increased her morbidity, innovative therapeutic alternatives are being searched for.

## References

[1] Romig T. Epidemiology of echinococcosis. Langenbecks Arch Surg. 2003;388(4):209–217. 10.1007/s00423-003-0413-3 12937989

[2] Akbulut S. Parietal complication of the hydatid disease: Comprehensive literature review. Medicine (Baltimore). 2018;97(21):e10671. 10.1097/MD.0000000000010671 29794743 PMC6392988

[3] Bhatnagar N, Kishan H, Sura S, Lingaiah P, Jaikumar K. Pelvic hydatid disease: A case report and review of literature. J Orthop Case Rep. 2017;7(4):4. 10.13107/jocr.2250-0685.834 PMC5702697 29181347

[4] Baumann S, Shi R, Liu W, et al. Worldwide literature on epidemiology of human alveolar echinococcosis: A systematic review of research published in the twenty-first century. Infection. 2019;47(5):703–727. 10.1007/s15010-019-01325-2 31147846 PMC8505309

[5] Song XH, Ding LW, Wen H. Bone hydatid disease. Postgrad Med J. 2007;83(982):536–542. 10.1136/pgmj.2007.057166 17675547 PMC2600110

[6] Nayman A, Guler I, Keskin S, et al. A novel modified PAIR technique using a trocar catheter for percutaneous treatment of liver hydatid cysts: A six-year experience. Diagn Interv Radiol. 2015;22(1):47–51. 10.5152/dir.2015.15011 PMC4712897 26574902

[7] Khan MS, Hashmi PM, Khan D. Eradication of advanced pelvic hydatid bone disease after limb salvage surgery – 5-year follow-up: A case report. J Med Case Reports. 2015;9(1):21. 10.1186/1752-1947-9-21 PMC4506416 26187499

[8] Notarnicola A, Panella A, Moretti L, Solarino G, Moretti B. Hip joint hydatidosis after prosthesis replacement. Int J Infect Dis. 2010;14:e287–e290. 10.1016/j.ijid.2009.12.006 20400352

[9] Kloppers C, Couzens-Bohlin K, Bernon M, et al. Hydatid disease in South-Africa – Is it a different disease in patients with HIV co-infection?. HPB. 2019;21:S593. 10.1016/j.hpb.2019.10.235

[10] Sidibé M. 2019 Global HIV statistics [homepage on the Internet]. UNAIDS. [cited 2022 June 7]. Available from: https://www.avert.org/global-hiv-and-aids-statistics

